# 离子液体固定化材料在固相萃取中的应用研究进展

**DOI:** 10.3724/SP.J.1123.2020.08002

**Published:** 2021-03-08

**Authors:** Yicong WANG, Leilei LIU

**Affiliations:** 吉首大学, 林产化工工程湖南省重点实验室, 湖南 张家界 427000; Key Laboratory of Hunan Forest Products and Chemical Industry Engineering, Jishou University, Zhangjiajie 427000, China; 吉首大学, 林产化工工程湖南省重点实验室, 湖南 张家界 427000; Key Laboratory of Hunan Forest Products and Chemical Industry Engineering, Jishou University, Zhangjiajie 427000, China

**Keywords:** 离子液体, 固定化, 固相萃取, 分子印迹聚合物, 综述, ionic liquid (IL), immobilization, solid phase extraction (SPE), molecularly imprinted polymer (MIP), review

## Abstract

离子液体是由阴、阳离子组成的低温熔融盐,几乎没有蒸汽压,具有稳定性好、溶解能力强、结构可设计、导电性好等优良性能。离子液体作为一种广受关注的新型“绿色溶剂”,具有代替传统有机溶剂的潜力,其制备方法和应用范围研究日趋完善和多样,已广泛应用于催化化学、光电化学、材料化学和分析化学等领域。离子液体通过功能化导向设计后,可以将羟基、氨基、羧基、氰基等活性基团键合在离子液体结构上,促使其更加易于与目标分子通过生成*π-π*键、氢键、离子键和范德华力等而产生相互作用,更加易于发生固定化反应。将离子液体负载到固体载体材料进行固定化后,新型材料既可以减少离子液体的流失,同时保留了离子液体和固体载体的独特性能,具有富集效率高、吸附容量高、稳定性好、识别位点多、萃取选择性强、离子液体利用率高等特点,近年来,在有机小分子固相萃取分离研究中应用广泛。该文从离子液体与硅胶、分子筛、分子印迹聚合物、氧化石墨烯、磁性材料等固体载体的固定化研究情况入手,综述了离子液体固定化材料在固相萃取分离中的应用情况,涉及的目标分离物质包括生物碱类、黄酮类、多酚类等天然活性成分,以及常见药物分子、有机农药等有机小分子化合物,系统地介绍了离子液体与多种载体固定化的性质、应用和分离机制。离子液体的引入,增加了复合材料的活性位点分布和吸附容量,离子液体固定化材料的吸附效率与离子液体种类、吸附材料用量、样品溶液浓度、吸附温度、pH值、洗脱溶剂类型、用量及流速等因素有关。该文探讨了离子液体结构相对单一、相关基础理论研究相对薄弱、复杂基质萃取程度不理想等问题,并提出相应的解决思路,以期为离子液体固定化材料在复杂基质中目标分子分离分析方面的应用提供借鉴和参考。

## Abstract (en)

Ionic liquids are low-temperature molten salts with almost no vapor pressure, and they are composed of organic cations and inorganic anions. Ionic liquids are characterized by the properties of good chemical stability, high solubility, designable structure, high conductivity and so on. The physicochemical properties of an ionic liquid depend on the nature and size of the cation and anion, which confer unique characteristics; hence, these reagents are also termed “designed extractants.” As a new class of green solvents, ionic liquids are potential replacements to traditional volatile organic solvents used for extraction; for this reason, ionic liquids have attracted the attention of scientists. Research on the methods of preparation and applications of ionic liquids is being diversified, and they are extensively used in catalytic chemistry, photoelectron chemistry, materials chemistry, analytical chemistry, etc. By functional guiding design, the structures of ionic liquids, especially the imidazole ring cations, can be easily grafted with active groups such as hydroxyl, amino, carboxyl, and cyano groups, so that interactions between the ionic liquids and target molecules can be promoted via the formation of *π-π* bonds, hydrogen bonds, ionic bonds, and van der Waals forces. In addition, ionic liquids can be readily immobilized on solid carriers by physical or chemical means in order to obtain a new solid material with ionic liquids embedded internally or decorated on the surface. Furthermore, ionic liquids could be converted into ionic liquid-immobilized composite materials by impregnation, grafting, etc. The resulting composites not only suffer minimal loss of ionic liquids but also retain the typical characteristics of the ionic liquids and solid materials, thus showing improved mass transfer performance and better adsorption performance. Immobilized materials are characterized by high enrichment efficiency, high adsorption capacity, good stability, and strong extraction selectivity, as well as the presence of numerous recognition sites and high utilization rate of ionic liquids. In recent years, they have been widely used as solid-phase extraction adsorption materials for the separation of small organic molecules. This review introduces common immobilization methods and the characteristics of ionic liquid-immobilized materials, as well as their application in solid-phase extraction. In this paper, methods for the immobilization of ionic liquids with solid carriers such as silica gel, molecular sieves, molecularly imprinted polymers, graphene oxide, and magnetic nanomaterials are summarized, and the application of ionic liquid-immobilized materials in solid-phase extraction is reviewed. The target substances include alkaloids, flavonoids, polyphenols, and other natural active components as well as common drug molecules, organic pesticides, and other organic small molecular compounds. The properties, applications, and separation mechanisms of ionic liquids immobilized with various carriers are systematically introduced. Literature survey shows that the distribution of the binding active sites of ionic liquid-immobilized materials to the target molecules is more uniform, which increases the adsorption capacity of the materials. The adsorption efficiency of ionic liquid-immobilized materials is related to the type of ionic liquid, amount of adsorption material, concentration of the sample solution, adsorption temperature, solution pH, flow rate of the eluent, and type and amount of the eluting solvent. The existing disadvantages of ionic liquids, such as simple structures, insufficient basic theoretical research, and unsatisfactory extraction degree in complex matrixes would also be discussed. The corresponding solutions would be presented with the aim of providing guidance for the application of ionic liquid-immobilized materials in the separation and analysis of targets in complex matrices, thus paving the way for a new direction in the field of extraction and separation.

随着绿色化学概念的提出,离子液体(ionic liquid, IL)作为一种新型绿色溶剂,因具有无蒸汽压、稳定性好、溶解能力强、结构可控、活性位点多等独特性质,在催化化学^[1]^、材料化学^[2]^和固相萃取^[3]^等领域受到的关注越来越多。进入21世纪,鉴于IL的可设计性,学者赋予了IL更多的功能性,开始设计合成一些具有独特性能的IL,如功能化IL^[4]^、手性IL^[5]^、聚合IL^[6]^、表面活性IL^[7]^、低共熔溶剂(deep eutectic solvents, DES)IL^[8]^等等。

IL阴、阳离子结构中可接枝-OH、-COOH、-NH_2_等活性基团,易与目标物之间发生*π-π*键、范德华力、氢键和静电等作用,有利于目标分子的吸附萃取,与传统试剂相比,展现出更高的效率。关于IL在分离领域的应用研究,主要体现在两个方面:(1)设计稳定性好、溶解能力强、黏度低的IL,添加至水、乙醇或甲醇等溶剂中,并联用超声波、微波、匀浆提取、红外加热等技术,用于有机化合物的提取分离,因IL具有较强的纤维素和半纤维素溶解能力,能够加速目标物质从细胞壁中溶出,特别适合于天然活性成分的提取^[9]^; (2)通过物理吸附或者化学键合方式,将IL固载于无机多孔材料、有机高分子材料、磁性纳米材料、分子印迹材料、石墨烯材料等固体材料上,制备成固相萃取剂,用于目标物的吸附分离,复合材料表面性质改变,兼具IL和固体载体特性,传质性能提高,具有更为优异的吸附解吸性能^[10]^。

IL黏度较高,限制了对目标分子的萃取,稀释使用又容易进入检测设备,流失严重。经固定化,IL虽然失去了液体性质,但依然保持了其独特的物理化学特性,有效克服了IL黏度大、不易操作等缺点。相对于其他固相萃取材料,IL固定化材料具有吸附容量高、pH稳定性好、识别位点多、萃取选择性强、IL利用率高等优点。自2009年Tian等^[11]^首次利用1-甲基咪唑和3-氯丙基三甲氧基硅烷研制出新型IL改性SiO_2_吸附剂,并用于丹参中丹参酮的固相萃取后,固相萃取的应用已是IL研究的一个全新领域,但相关研究起步较晚,主要用于萃取分离常见有机小分子。另外,IL固定化后,增大了目标物质与IL的接触面积,对痕量有机污染物的分离富集效果突出。本文对近些年IL固定化技术的分离应用研究进行了详细综述,系统地介绍了其在有机小分子固相萃取中的应用进展情况。

## 1 离子液体固定化

离子液体在各领域中的使用,一直存在着成本高、黏度大、回收困难、与目标物分离困难、流失严重等问题。近些年,如何将IL固定化,减少其泄露,成为众多学者努力研究的方向。所谓IL固定化是指通过物理或化学方法将IL固定于固体载体上,从而得到一种表面含有IL结构或者包埋IL液膜的新型固体材料,既节约IL的用量又提高其利用率。新材料既在一定程度上保留了固体载体的特性,又具有IL的特性,更加便于应用。常用的IL固体载体分为无机和有机材料,前者包括硅胶、活性炭、分子筛和珍珠岩等,后者包括聚苯乙烯、大孔树脂、聚醚微球、分子印迹聚合物等高聚物。

常用的IL固定化方法主要有液膜法、浸渍法、接枝法、溶胶-凝胶法等^[12]^。其中液膜法主要是将IL通过涂布、浸渍和加压填充等方式,与有机聚合物或无机多孔膜等材料形成IL支撑液膜或IL聚合物膜,使得IL在一定固体形态范围内活动,降低了其流动性,利于物质的分离,Lee等^[13]^采用多相分离技术将IL与聚偏氟乙烯基体结合,制备出IL支撑液膜,用于天然气中酸性气体的脱除,分离性能良好。浸渍法是将IL滴加至固体载体上至完全湿润,或者将载体完全浸润至过量的IL中,然后以索氏抽提法除去多余的IL,操作相对简单。浸渍法对载体破坏较小,但适用范围较窄,仅对小部分载体可发生作用,对大部分载体无法作用,其作用可以是物理吸附,也可以是载体和IL活性基团之间形成特定共价键产生化学吸附。例如,蔡志锋等^[14]^将1-(4-丁基磺酸)-3-甲基咪唑硫酸氢盐([(*n*-Bu-SO_3_H)MIm][HSO_4_])溶解在乙醇中,然后缓慢加入纳米SiO_2_载体,FT-IR显示酸性IL成功固定在SiO_2_表面,且IL引入未改变SiO_2_的结构。DeCastro等^[15]^将IL滴加到SiO_2_载体上,在制备路易斯酸型咪唑IL固定化材料过程中发现有HCl气体生成,采用固体核磁共振波谱(^29^Si MAS NMR)对固定化IL进行表征时发现,(SiO)_2_Si-OH和(SiO)_3_Si-OH中的Si-OH基团在-91和-101处的化学位移值消失,说明IL阴离子与硅胶上的硅烷醇反应生成了共价键。

因为多数载体属于惰性材料,性质稳定,不易与IL直接发生作用,需要先在载体或IL阳离子上键入能相互发生反应的功能性基团,如-OH、-COOH、-NH_2_、-CN等,然后再进行固定化反应,称之为接枝法。当以SiO_2_为载体时,一般需先活化硅胶,然后用3-氯丙基三乙氧基硅烷^[16]^、3-巯基丙基三甲氧基硅烷^[17]^或乙烯基三乙氧基硅烷^[18]^在硅胶上接枝氯丙基、巯基丙基或乙烯基基团。以Fe_3_O_4_粒子为载体制备磁性材料时,因其自身易被氧化,有强烈的聚集倾向,易发生沉淀,需要通过偶联剂、表面活性剂、生物大分子或碳包埋等方式进行修饰接枝,以降低其表面自由能,增强其稳定性和生物相容性。溶胶-凝胶法是将一些功能单体混合均匀,进行水解、缩合反应,形成稳定透明的溶胶体系,然后胶粒间缓慢聚合形成多维网络结构的凝胶,经干燥烧结制成纳米材料,在溶胶-凝胶的过程中将IL包埋在凝胶颗粒的网络结构中,如IL代替常规溶剂,作为溶剂、致孔剂、模板剂或功能单体,制备成分子印迹聚合物,为分子印迹技术的识别分离应用注入了新活力^[19]^。

## 2 离子液体固定化材料在固相萃取中的应用

### 2.1 硅胶为载体

硅胶是一种高活性吸附材料,机械稳定性好,表面含有大量的微孔,应用范围极广。硅胶的IL固定化方法主要以接枝法为主,首先用硝酸溶液活化SiO_2_,以增强SiO_2_表面硅醇基团的含量,除去金属氧化物和含氮杂质;然后添加3-氯丙基三乙氧基硅烷等,制备氯丙基SiO_2_;最后放置于甲苯中回流,生成IL硅胶材料。将IL负载于硅胶表面后,IL用量减少,传质速率提高,回收率增大,已广泛应用于天然产物的固相萃取,已知适合IL硅胶材料萃取分离的天然活性成分包括萜类、多糖、生物碱、多酚等类型(见表1)。

**表1:** IL硅胶材料在固相萃取中的应用

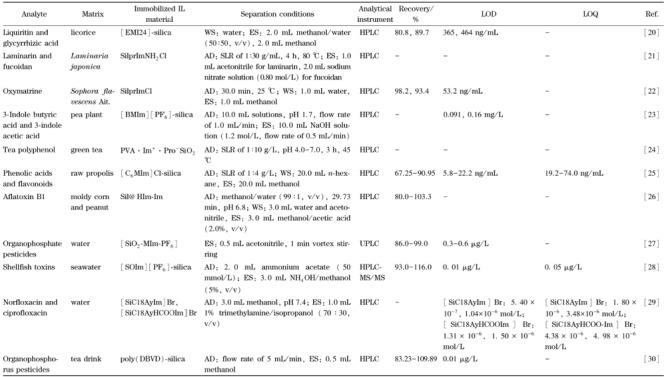

AD: adsorption; WS: washing solvent; ES: elution solvent; SLR: solid-liquid ratio; -: no data; [EMI24]: 2-ethyl-4-methylimidazole; SilprTmNH_2_Cl: 1-( 3-aminopropyl ) imidazolium chloropropyl SiO_2_ material; SilprImCl: imidazole chloropropyl SiO_2_ material; [BMIm][PF_6_]: 1-butyl-3-methylimidazolium hexafluorophosphate; PVA . Im^+^ · Pro^-^ SiO_2_: polyvinyl alcohol N-methylimidazolium proline SiO_2_ material; [C_6_MIm ] Cl: 1-hexyl-3-methylimidazolium chloride; Sil @Hlm-Im: imidazolium chloride-butylimidazolium chloride-based silica; [SiO_2_-MIm-PF_6_]: methyl imidazole hexafluorophosphate SiO_2_ material; [SOIm] [PF_6_]: octyl imidazole hexafluorophosphate; [SiC18AyIm ] Br: 3-octadecyl- 1-allylimidazole bromide SiO_2_ material; [SiCl8AyHCOOm]Br: 3-octadecy1-1-*H*-imidazole-l-carboxylate bromide SiO_2_ material; poly(DBVD): poly(3,3'-dodecane-1 , I'-bis- 1-vinylimidazolium dibromide; HPLC: high efficiency liquid chromatography; UPLC: ultra performance liquid chromatography; MS: mass spectrum; LOD: limits of detection; LOQ: limits of quantification.

Tian等^[20]^合成了2-乙基4-甲基咪唑基SiO_2_吸附剂,固相萃取甘草中甘草素和甘草酸;与传统的C18吸附剂相比,IL型SiO_2_材料对分析物亲和力较高,选择性更高,目标分子的吸附量分别达0.18 mg/g和1.0 mg/g。Zhang^[21]^等基于氯丙基SiO_2_ (SilprCl),通过多步合成法制备出咪唑氯丙基SiO_2_ (SilprImCl)、1-丁基咪唑氯丙基SiO_2_ (SilprBimCl)、2-丙基咪唑氯丙基SiO_2_ (SilprPImCl)和1-(3-氨基丙基)咪唑氯丙基SiO_2_ (SilprImNH_2_Cl),用于海带多糖的固相萃取;结果发现不同材料对岩藻多糖的吸附顺序为:SilprImNH_2_Cl >商用NH_2_固相萃取柱>SilprImCl>SilprBImCl>SilprPImCl,对海带多糖的吸附顺序为:SilprBImCl>SilprPImCl>商用NH_2_固相萃取柱>SilprImCl>SilprImNH_2_Cl,其中SilprImNH_2_Cl中阴离子易于发生置换反应,亲水性强,吸附岩藻多糖能力强,吸附海带多糖能力较低,海带多糖更容易被洗脱,最终岩藻多糖的吸附量为54.18 mg/g,海带多糖的吸附量为55.10 mg/g。

Bi等^[22]^先制备氯丙基硅胶,然后合成了8种IL硅胶材料:SilprImCl、1-甲基咪唑氯丙基SiO_2_ (SilprMImCl)、1-乙基甲基咪唑氯丙基SiO_2_ (SilprEImCl)、SilprBImCl、氟硼酸咪唑氯丙基SiO_2_ (SilprImBF_4_)、六氟磷酸咪唑氯丙基SiO_2_ (SilprImPF_6_)、双三氟甲烷磺酸亚胺咪唑氯丙基SiO_2_ (SilprImTf_2_N)和氨丙基SiO_2_ (SilprNH_2_),从苦参中提取分离苦参碱和氧化苦参碱;实验发现,从SilprImCl至SilprBimCl,烷基链长度增加,IL氢键酸度和疏水作用增加,限制了IL硅胶与目标分子的作用,吸附量减少,SilprImCl吸附量最高;同时,比较含4种阴离子(Cl^-^、B $F_{4}^{-}$ 、Tf_2_N^-^和P $F_{6}^{-}$ )的IL硅胶材料,发现SilprImCl在水中的pH值相对较低,有助于吸附,吸附量较高,另外,SilprImCl重复利用效果最好,经4次循环使用后,对氧化苦参碱的回收率仅从93.4%降低至89.7%,下降较少。Sheikhian等^[23]^先用硝酸溶液活化硅胶,制备出1-丁基-3-甲基咪唑六氟磷酸([BMIm]PF_6_)SiO_2_材料,从豌豆中萃取富集3-吲哚丁酸和3-吲哚乙酸;实验发现目标分子萃取率受pH值影响很大,而水相离子强度受温度影响较小,以NaOH溶液进行洗脱,可重复使用近10次,2种激素的富集因子分别达到了100和4000。Zhang等^[24]^基于*N*-甲基咪唑脯氨酸盐和聚乙烯醇链基硅胶,合成了一种新型仿生多触手IL硅胶(聚乙烯醇*N*-甲基咪唑脯氨酸盐SiO_2_, PVA·Im^+^·Pro^-^@SiO_2_),固相萃取绿茶中茶多酚;结果发现,该新型材料具有吸附量高、重复利用性好的特点,对目标物选择性好,茶多酚的吸附容量可达236.84 mg/g。2014年,Wang等^[25]^采用浸渍法制备IL-SiO_2_材料,通过IR检验IL硅胶材料结构稳定性,结合基质固相分散技术萃取生蜂胶中8种酚酸和黄酮;结果发现3种IL硅胶材料的萃取能力顺序为:1-丁基-3-甲基咪唑氯盐([BMIm]Cl)≈1-己基-3-甲基咪唑氯盐([C_6_MIm]Cl)>1-辛基-3-甲基咪唑氯盐([C_8_MIm]Cl), [C_8_MIm]Cl与目标分子氢键作用较强,洗脱困难,[BMIm]Cl在色谱图中有干扰峰,影响咖啡酸和白杨素的分析,含[C_6_MIm]Cl材料的吸附效果优于索氏提取和超声辅助提取。

IL负载于硅胶后,对目标分子识别能力增强,选择性好,特别适合于痕量有机污染物的萃取分离。Fang等^[26]^以硅胶为载体,通过接枝法合成了8种新型双功能吸附剂,即咪唑氯化丁基咪唑氯化二氧化硅(Sil@BIm-Im)、甲基咪唑氯化丁基咪唑氯化二氧化硅(Sil@BIm-MIm)、乙基咪唑氯化丁基咪唑氯化二氧化硅(Sil@BIm-EIm)、双丁基咪唑氯化二氧化硅(Sil@BIm-BIm)、氯化咪唑己基咪唑氯化二氧化硅(Sil@HIm-Im)、甲基咪唑氯化己基咪唑氯化二氧化硅(Sil@HIm-MIm)、乙基咪唑氯化己基咪唑氯化二氧化硅(Sil@HIm-EIm)和丁基咪唑氯化己基咪唑氯化二氧化硅(Sil@HIm-BIm),用于固相萃取发霉花生和玉米中黄曲霉毒素B1;通过响应曲面优化甲醇/水比例、时间、pH值等吸附条件,发现Sil@HIm-Im为最佳吸附剂,发霉玉米和花生中黄曲霉素B1的含量分别为0.009和0.023 μg/g。

Galán-Cano等^[27]^合成氯化甲基咪唑硅胶材料[SiO_2_-MIm-Cl],然后加入试剂KPF_6_,通过置换反应转化合成[SiO_2_-MIm-PF_6_],作为分散微小固相萃取剂富集水样中的有机磷杀虫剂;发现[SiO_2_-MIm-Cl]在水中容易解离,对目标分子吸附效果较差,但NaCl的加入能限制材料的解离作用,吸附能力略有增加;[SiO_2_-MIm-PF_6_]疏水性高,与分析物的相互作用更强,有利于目标分子的吸附,但加入NaCl后,导致[SiO_2_-MIm-PF_6_]形成困难,萃取率降低。孙晓杰等^[28]^将辛基功能化IL接枝到硅胶表面,制备出辛基咪唑六氟磷酸盐键合硅胶材料(Silica-[SOIm][PF_6_]),并填制固相萃取柱,结合高效液相色谱质谱联用(HPLC-MS/MS)对海水中3种贝类毒素进行富集检测;该材料主要通过强疏水作用和弱离子交换作用吸附目标分子,其萃取性能与具有亲脂亲水性能的商用HLB柱相当。Passos等^[29]^基于1-烯丙基咪唑和烯丙基1-*H*-咪唑-1-羧酸盐,通过多步合成法合成了2种C18硅胶固定化IL[SiC18AyIm]Br和[SiC18AyHCOOIm]Br,经紫外光照射无害化处理后,用于诺氟沙星、环丙沙星的固相萃取性能测试;经*t*检验统计分析后发现,不同的材料吸附不同化合物没有显著性差异,[SiC18AyHCOOIm]Br对诺氟沙星具有较高的保留率。

双咪唑离子液体中带有两个咪唑环,相比于单咪唑离子液体,能提供更多的作用位点及更多种类的作用力。杨振等^[30]^通过自由基聚合反应,将3,3'-十二烷-1,1'-二-1-乙烯基双咪唑溴盐(DBVD)与乙烯基三乙氧基硅烷修饰的硅胶共价聚合,得到聚合双咪唑离子液体硅胶材料Poly(DBVD)-Sil,用于固相萃取茉莉蜜茶和茉莉花茶饮料中的3种农药喹硫磷、倍硫磷和辛硫磷;考察吸附剂的用量、上样流速、解吸剂的种类和用量后,发现该方法的LOD达0.01 μg/L,样品基质无干扰。

开发高级功能化色谱固定材料对促进色谱分离科学的发展具有重要意义。Zhou等^[31]^通过表面自由基链转移反应,将奎宁键合在SiO_2_表面合成聚奎宁二氧化硅(Sil-PQn),然后以2-氯乙基异氰酸酯为桥接分子,通过亲核取代反应,用*N*-甲基咪唑对Sil-PQn进行改性,制备新型咪唑IL功能化聚奎宁改性SiO_2_材料(Sil-PQn-MIm),合成过程如图1所示,并填制色谱柱,采用芳香酸、核苷/核酸酶、磺胺类药物、烷基苯、苯和多环芳烃等多种试剂,测试Sil-PQn-MIm色谱柱的多模式色谱性能;发现含IL的Sil-PQn-MIm柱具有更好的分离性能,重现性较好,RSD达0.15%~0.72%,理论塔板数高达90000板/米。Song等^[32]^首先通过氨基IL与柠檬酸反应合成咪唑离子液体衍生碳点(ImCDs),然后将其接枝到氨基丙基修饰的SiO_2_表面,制备新型固定相Sil-ImCDs;同时用碱基、核苷、氨基酸、糖类和人参皂苷进行色谱性能考察,发现咪唑离子液体和柠檬酸形成碳点后存在丰富的官能团,Sil-ImCDs具有更好的亲水相互作用;对比商用的Sil-MIm和Sil-NH_2_色谱柱后,发现Sil-ImCDs具有更好的分离效果。

**图1:**
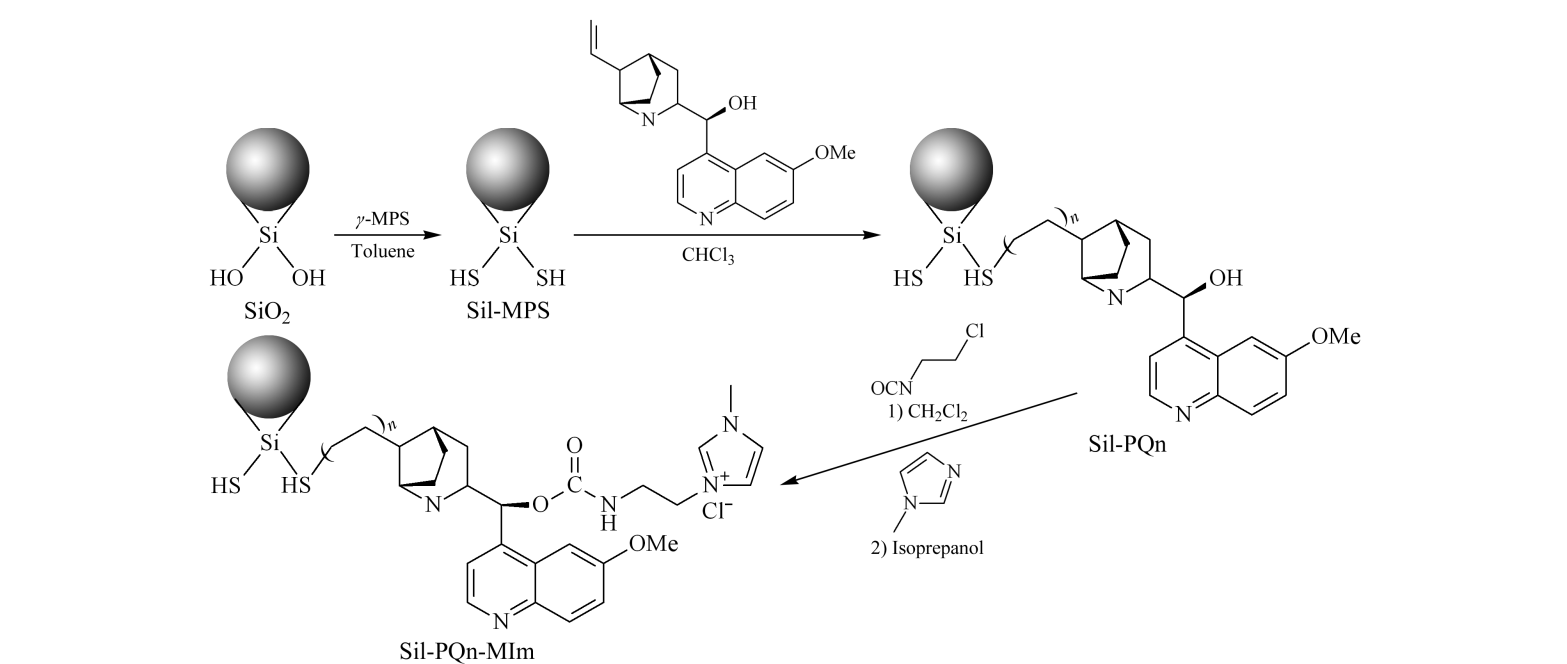
Sil-PQn-MIm的制备过程示意图^[31]^

DES是一种稳定性更高、降解容易、成本低廉的新型IL,一般是由一定化学计量比的氢键受体(如季铵盐)和氢键给体(如羧酸、醇和胺类)组合而成的低共熔混合物,具有较强的*π-π*键及氢键生成能力,应用广泛。Gu等^[33]^基于氯化胆碱(ChCl)和尿素,按物质的量之比1∶2混合制备DES,选择5种硅烷化试剂,对球形多孔SiO_2_进行表面改性,制备*N*-甲基-葡萄糖胺功能化的硅胶色谱固定相,色谱分离性能考察发现:该硅胶材料对人参皂苷、核苷和磺胺类物质具有良好的分离效率和选择性,与有机溶剂相比,DES的加入增加了二氧化硅的分散性。Hu等^[34]^基于ChCl和乙二醇DES自由基聚合反应,制备了2种共聚物,即聚(甲基丙烯酸二甲氨基乙酯/衣康酸)改性SiO_2_(Sil-PDM-PIA)和聚(甲基丙烯酸二甲氨基乙酯/丙烯酸)改性SiO_2_(Sil-PDM-PAA),制成色谱柱,用于分离核苷、碱基和糖类,发现这两种新型的共聚物相比于聚(衣康酸)改性SiO_2_(Sil-PIA)和聚(丙烯酸)改性SiO_2_(Sil-PAA),在HILIC中具有更高的选择性和极性分离能力,且Sil-PDM-PAA的选择性和色谱分离性能明显优于Sil-PDM-PIA。Li等^[35]^以ChCl和甘油为原料,按物质的量之比1∶1至1∶5合成了5种DES,用于从麦麸中提取阿魏酸,其中ChCl和甘油的物质的量之比为1∶2混合制备的DES提供了最高的提取率;然后用5种DES对SiO_2_进行改性,并将其与*N*-甲基咪唑IL改性硅胶和硅胶进行比较,发现改性硅胶表现出较高的亲和力,萃取效率最高。

### 2.2 聚合物为载体

IL聚合物材料在固相萃取中的应用见表2。其中酚醛树脂具有孔隙率高、热稳定性好、原料成本低等特点,已被广泛应用于分离领域。Li等^[36]^以3-氨基苯酚作为功能单体,乙醛酸作为绿色交联剂,聚乙二醇6000作为致孔剂,合成咪唑IL改性酚醛树脂,并结合HPLC用于提取和检测黄瓜中微量植物激素噻二唑仑和氯芬隆;发现因咪唑IL提供了更多的相互作用位点,IL修饰的酚醛树脂比未修饰的吸附效果更好。

大孔树脂是以苯乙烯和丙酸酯为单体,乙烯苯为交联剂,甲苯和二甲苯为致孔剂,经过相互交联聚合形成的多孔高分子聚合物。黄良樑等^[37]^将[BMIm]BF_4_固定到大孔树脂上,制成固相萃取小柱,结合*β*-环糊精(*β*-CD)修饰IL碳糊电极,电化学检测水样中痕量双酚A;发现IL与*β*-CD发生了包合作用,能够排除主要的样品基质干扰,双酚A易于被亲水性IL通过氢键作用和*π-π*键作用富集到电极表面,选择性识别和响应增强;实际样品分析显示该方法与SPE-HPLC-UV标准方法无显著性差异。Raoov等^[38]^制备出新型*β*-CD-IL大孔聚合物材料(*β*-CD-BIMOTs-TDI),结合GC-FID,用于测定河水中6种多酚;与*β*-CD-甲苯2,4-二异氰酸酯(TDI)、聚苯乙烯二乙烯基苯吸附树脂(HR-P)、聚苯乙烯二乙烯基苯吸附树脂(HR-X)、C18硅胶比较发现,*β*-CD-BIMOTs-TDI回收率最高,达103%~114%,远远高于*β*-CD-TDI的32%~57%,新型*β*-CD-IL材料与酚类之间主要通过强的*π-π*键和形成包合物发生作用,同时IL的存在提高了酚类对*β*-CD孔腔的选择性,而*β*-CD-TDI与多酚之间仅有弱的包合作用,吸附较弱。

**表2:** IL聚合物材料在固相萃取中的应用

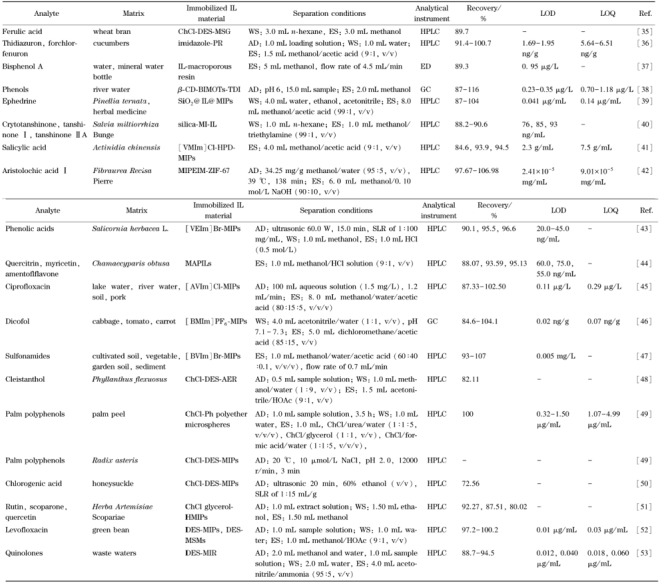

ChCl: choline chloride; MSG: modified silica gel; PR: phenolic resin; β-CD-BIMOTs- TDI: β-cyclodextrin-IL polymers; CD: cyclodextrin; TDI: toluene diisocyanate; MI: imidazole; [VMIIm] Cl: 1-vinyl-3-( 2-methoxy-2-oxyethyl) imidazole chloride; HPDMIPs: hollow porous dummy MIPs; MIPEIM: MIP composited ethylimidazole IL; ZIF-67: zeolitic imidazolate frame-work-67; [VEIm ] Br: 1-vinyl-3-ethyl imidazole bromide; MAPIIs: molecularly imprinted anion-functionalized poly( IL)s; [AVIm]Cl: 1-allyl-3-vinyl imidazole chloride; [BVIm] Br: 1-butyl-3-vinyl imidazole bromide; AER: anion exchange resin; Ph: benzene; HMIPs: hybrid molecularly imprinted polymers; MSMs: mesoporous siliceous materials; MIR: molecularly imprinted resin; GC: gas chromatography; ED: electrochemical detection.

分子印迹聚合物(molecularly imprinted polymers, MIPs)对目标分子具有特定空间结构和特定识别位点,制备过程中,模板分子在MIPs中的位置会被“记忆”下来,洗去模板后,MIPs表面形成了一个“空穴”,分离时与模板分子结构相似的目标物质会被保留在该“空穴”中,从而实现目标分子的精准分离。IL相比常规有机溶剂,溶解性能更佳,将IL应用于分子印迹技术从而制备出IL基MIPs材料,结构中因IL活性基团的存在,增强了其识别性能,进一步拓展了MIPs的应用,例如以硅胶、分子筛等为固体辅助载体制备的MIPs在天然活性成分固相萃取中的应用。

Fang等^[39]^首先合成氯丙基硅胶,然后,将咪唑IL负载于硅胶上,随后添加交联剂甲基丙烯酸缩水甘油酯(GMA)和乙二醇二甲基丙烯酸酯(EGDMA),以及模板剂麻黄碱,通过5步反应制备而成SiO_2_@IL@MIPs(其制备示意图如图2所示),从半夏、10种常见药物和尿液样品中萃取分离麻黄碱;发现以甲醇/乙酸(99∶1, v/v)作为洗脱溶剂效果最好;半夏中的麻黄碱为5.50 μg/g, 10种草药中的麻黄碱为0~46.50 μg/g,尿液中的为68.70~102.80 μg/mL;对比硅胶、SiO_2_@IL-NH_2_、SiO_2_@IL@NIPs(无模板IL印迹材料)和SiO_2_@IL@MIPs 4种吸附材料,结果表明含IL基团的MIPs与麻黄碱的相互作用较强,吸附量显著增加。Tian等^[40]^以9,10-菲二酮作为模板分子,引入分子印迹技术,合成分子印迹IL改性SiO_2_(MI-IL-silica),作为SPE吸附剂,用于固相萃取分离中药丹参中隐丹参酮、丹参酮Ⅰ和丹参酮ⅡA;对比IL-SiO_2_、商用SiO_2_和C18材料的效率,发现该分子印迹IL改性SiO_2_材料具有更高的选择性,可以从功能性饮料中直接选择性分离3种目标丹参酮。

**图2:**
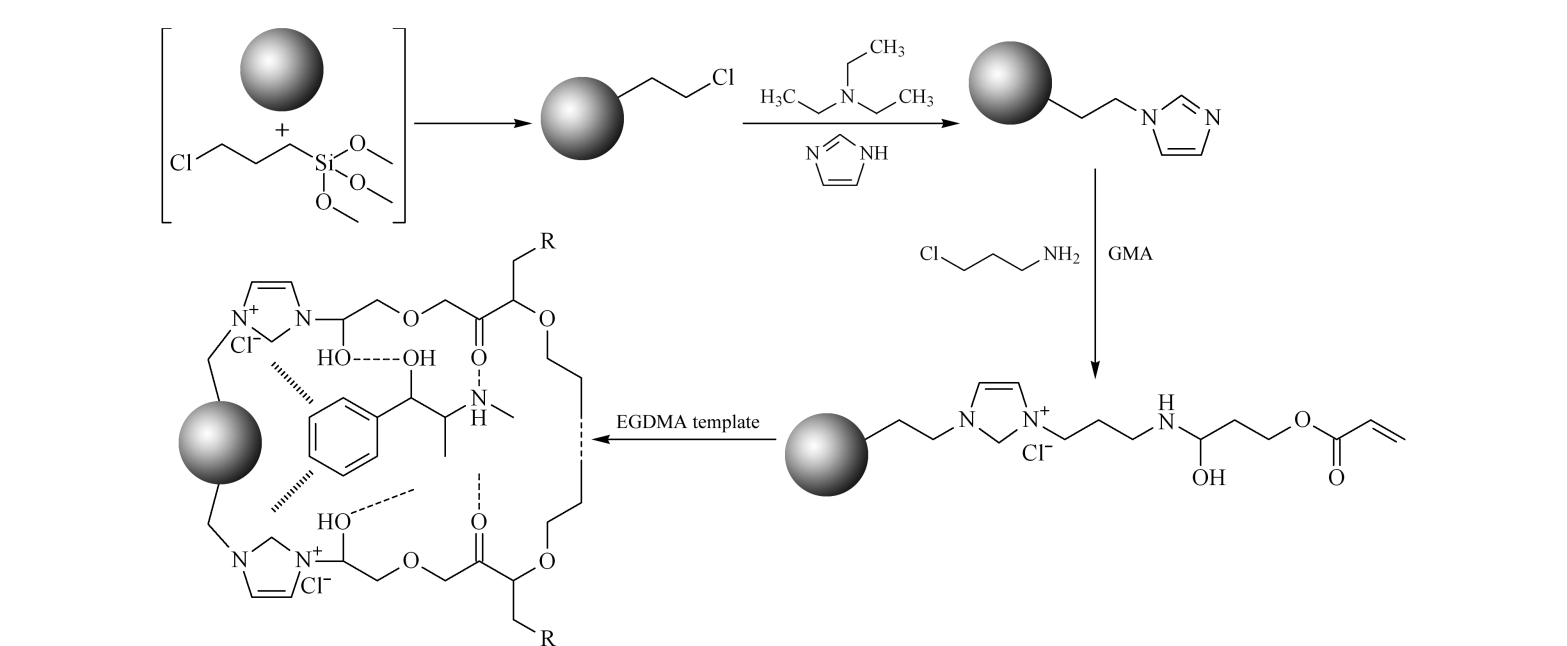
SiO_2_@IL@MIPs的制备过程^[39]^

水杨酸存在强分子内氢键,能够削弱其与功能单体分子间的氢键。为制备具有高容量和良好选择性的MIPs, Xiang等^[41]^选择以苯甲酸为模板、1-乙烯基-3-丁基咪唑氯盐([VMIm]Cl)为功能单体、分子筛MCM-48作为载体,制备多孔分子印迹聚合物HPDMIPs,并从猕猴桃中选择性萃取水杨酸,回收率达84.6%~94.5%;实验发现,HPDMIPs是一种高比表面积和空心多孔结构材料,具有较高的结合能力、选择能力和更快的水杨酸传质性能;由于猕猴桃中水杨酸含量较低,经过HPDMIPs吸附分离后,色谱分析中未出现相关峰,因此配制水杨酸含量为0.18 g/g的鲜猕猴桃样品验证HPDMIPs的吸附性能。Fang等^[42]^以马兜铃酸I为模板,基于氨基乙基咪唑IL、ZIF-67沸石和分子印迹聚合物,经过多步合成制备了IL基分子印迹聚合沸石材料(MIPEIM-ZIF-67),用于固相萃取分离马兜铃酸I;与无印迹的IL基ZIF-67聚合物对比发现,MIPs中模板有其特定空间结构,可比其他吸附剂吸附更多的目标物;在较高温度下,较高的分子内能量会导致分子间作用力减弱,目标物吸附量随着温度的升高而降低,优化参数后,马兜铃酸I的吸附量为0.043 mg/g。

Bi等^[43]^基于4种IL,即1-烯丙基-3-乙基咪唑溴盐([AEIm]Br)、1-烯丙基-3-辛基咪唑氯盐([AOIm]Cl)、1-烯丙基-3-己基咪唑氯盐([AC_6_Im]Cl)和1-烯丙基-3-丁基咪唑氯盐([ABIm]Cl)制备分子印迹阴离子交换聚合物,用于从玻璃麦草中固相萃取3种酚酸;实验发现聚合物对酚酸的吸附顺序为:阿魏酸>咖啡酸>原儿茶酸,回收率分别为95.5%、96.6%和90.1%; IL结构对聚合物吸附容量有显著影响,烷基链长度从乙基增加至辛基,疏水相互作用增加,IL氢键酸度增加,封堵了聚合物的孔隙,对酚酸的吸附性能提高,如果官能团之间的距离太近,受空间位阻效应影响,目标化合物不能有效地与IL官能团相互作用;同时IL阴离子交换聚合物可通过阴离子置换反应再生。Bi等^[44]^选择以槲皮素为模板,咪唑IL为功能单体,EDGMA为交联剂,采用热引发聚合法制备了分子印迹聚合物(MAPILs),用于日本扁柏中槲皮素、杨梅素和穗花杉双黄酮的多相分散萃取,同时选择多种阴离子,通过阴离子复分解法对聚IL-MIPs进行功能化处理,以提高目标黄酮分离效率;目标化合物通过样品-溶剂-吸附剂三相进行固体分散萃取,然后再去除样品基质并清洗;该方法显著降低了杂质干扰和分析成本,参数优化后,3种黄酮的回收率分别达到88.07%、93.59%和95.13%。

IL-MIPs因特有的分子识别能力,已广泛应用于痕量有机分子残留的固相萃取中。Zhu等^[45]^以1-烯丙基-3-乙烯基咪唑氯盐([AVIm]Cl)和2-甲基丙烯酸羟乙酯设计开发出一种亲水性环丙沙星印迹聚合物,功能单体中含有丰富的亲水基团,如-Cl、-OH、-C=O等,促使聚合物材料与目标物之间容易发生氢键作用、静电相互作用和*π-π*偶极相互作用,形成了很强的亲和力,同时避免了水分子的非特异性吸附;该材料对水基质中喹诺酮类抗生素环丙沙星、左氧氟沙星和甲磺酸培氟沙星,具有特殊的分子识别作用,对比发现新建方法与标准方法GB/T 21312-2007之间没有显著性差异。Yan等^[46]^以[BMIm]PF_6_为辅助溶剂,以*α*-Cl-DDT为虚拟模板,采用沉淀聚合法制备了新型IL分子印迹聚合物(IL-MIPs),用于从卷心菜、番茄和胡萝卜中快速筛选三氯杀螨醇;对比发现,IL-MIPs聚合物对三氯杀螨醇的吸附能力和选择性远高于IL非印迹聚合物和非印迹聚合物。Zhu等^[47]^为了提高分子印迹聚合物在强极性溶剂中的选择性吸附性能,基于1-乙烯基-3-正丁基咪唑溴盐([VBIm]Br)和分子印迹技术研制了一种新型磺胺甲恶唑IL基分子印迹聚合物;静态平衡实验表明,得到的IL分子印迹聚合物对甲醇溶液中磺胺甲恶唑、磺胺甲基甲氧嘧啶和磺胺嘧啶的分子识别能力较强,对二苯胺、甲硝唑、2,4-二氯苯酚和间二羟基苯等干扰物的吸附相当低,有利于萃取;^1^H NMR分析表明,IL分子印迹聚合物的优异识别性能主要基于IL基团容易与磺胺类分子形成氢键、静电和*π-π*电荷等相互作用。

阴离子交换树脂(AER)是一种固相萃取吸附剂,结构中含有胺或季铵盐基团,比表面积高,活性官能团多,可提供更多的活性中心与相反电荷的自由离子相互作用。Gan等^[48]^利用AER和ChCl-DES,改性制备了DES-AER复合材料,用于萃取分离落萼叶下珠中cleistanthol,对比3种吸附材料ODS、AER、DES-AER发现DESs改性使树脂表面更加粗糙,微孔变多,有利于目标分子的吸附解析;DES-AER对cleistanthol的吸附容量达0.671~1.842 mg/g,远高于AER的0.154~1.016 mg/g,同时亲和选择性更显著,cleistanthol回收率达82.11%,高于另外2种吸附材料。付静娜等^[49]^制备出8种ChCl-DES聚醚微球材料,用于分离纯化原儿茶酸、儿茶素、表儿茶素和咖啡酸;先考察DES的提取效果,再考察聚醚微球材料的效果,结果发现8种DESs的萃取效果优于甲醇,ChCl-甲酸DES物质的量之比1∶1的效果最好;固相萃取中,由于ChCl-Ph DES修饰的聚醚微球表面粗糙,同时结构中苯环与多酚的相互作用更强,其萃取效果最好。

Li等^[50]^基于ChCl合成DES,结合分子印迹技术制备出DES-MIPs,从金银花中固相萃取分离绿原酸,对比不含DES和绿原酸模板的MIPs材料,发现MIPs中键合DES后,能够提高材料的亲和力、选择性和吸附能力,对绿原酸具有特异识别能力;DES-MIPs、DES-NIPs、MIPs和NIPs(不含模板印迹材料)对绿原酸的SPE回收率分别为72.56%、64.79%、69.34%和60.08%,其中DES-MIPs最高,绿原酸的吸附量达12.57 mg/g。Li等^[51]^同时以芦丁、滨蒿内酯和槲皮素为模板,基于ChCl-DESs、1-乙基-3-甲基咪唑溴盐([EMIm][Br])、1-丁基-3-甲基咪唑溴盐([BMIm][Br])和1-己基-3-甲基咪唑溴盐([HMIm][Br])制备出IL分子印迹聚合物,用于固相萃取青蒿中芦丁、滨蒿内酯和槲皮素;发现基于DES修饰的HMIPs的回收率高于IL,其中选择DES-2-HMIPs进行萃取时,色谱图干扰峰最低,形状最佳,同时与无模板DES-HMIPs相比,发现DES-2-HMIPs的表面具有团聚性和不规则性,其表面粗糙、多孔、松散,松散的结构不仅减少了目标分子从流动相到固定相的传质阻力,也增加了HMIPs的比表面积,有利于将芦丁、滨蒿内酯和槲皮素包埋到DES-2-HMIPs的孔腔中,更容易吸附和解吸,结果萃取得到的目标分子含量分别为5.6 、2.3和3.4 mg/g。付娜静等^[49]^以ChCl-咖啡酸(CA)-乙二醇(EG)三元低共熔溶剂为功能单体,以咖啡酸为模板剂,制备出13种分子印迹材料ChCl-CA-EG DES MIPs,用于识别4种多酚物质;与NIP、C18、C8进行比较,发现制备的MIP-5聚合物具有良好的特异性识别能力,能高效识别中药祁紫菀中的多酚,同时与合成材料MIP-2、MIP-3、MIP-4相比,其吸附量随着模板量增加而增加,结果表明模板量与交联剂物质的量之比为1∶3、吸附360 min、20 ℃、pH 2、氯化钠10 mmol/L时,多酚吸附量最大,特异性识别能力最强。

Li^[52]^采用加热法合成了甜菜碱基DES,并对MIPs和介孔硅质材料(MSMs)进行改性(其制备示意图如图3所示),研制了6种固相萃取吸附剂,对左氧氟沙星进行快速纯化;其中DES-MIPs表现出比传统材料更好的选择性吸附能力,对左氧氟沙星具有更好的分子识别能力和结合能力,发现DES-MIPs对绿豆提取物中左氧氟沙星的回收率高达95.2%,能有效地去除干扰物质;而介孔材料DES-MSMs也表现出优于传统MSMs的吸附容量。Tang等^[53]^以富含亲水基团-OH、-NH_2_、-NH-的间苯二酚和三聚氰胺作为功能单体,筛选了ChCl-乙二醇、四甲基溴化铵-乙二醇和四甲基氯化铵-乙二醇等3种DES,合成水溶性DES基分子印迹树脂,用于固相萃取水中喹诺酮类药物;研究发现,传统的分子印迹树脂MIR表面相对粗糙,而DES-MIR表面是一种粗糙多孔的结构,可以为目标分子提供更具体的识别位点,不同的DES-MIR聚合物具有不同的吸附容量,其中环丙沙星和氧氟沙星在DES-1-MIR上吸附容量最高,达32.92 mg/g。

**图3:**
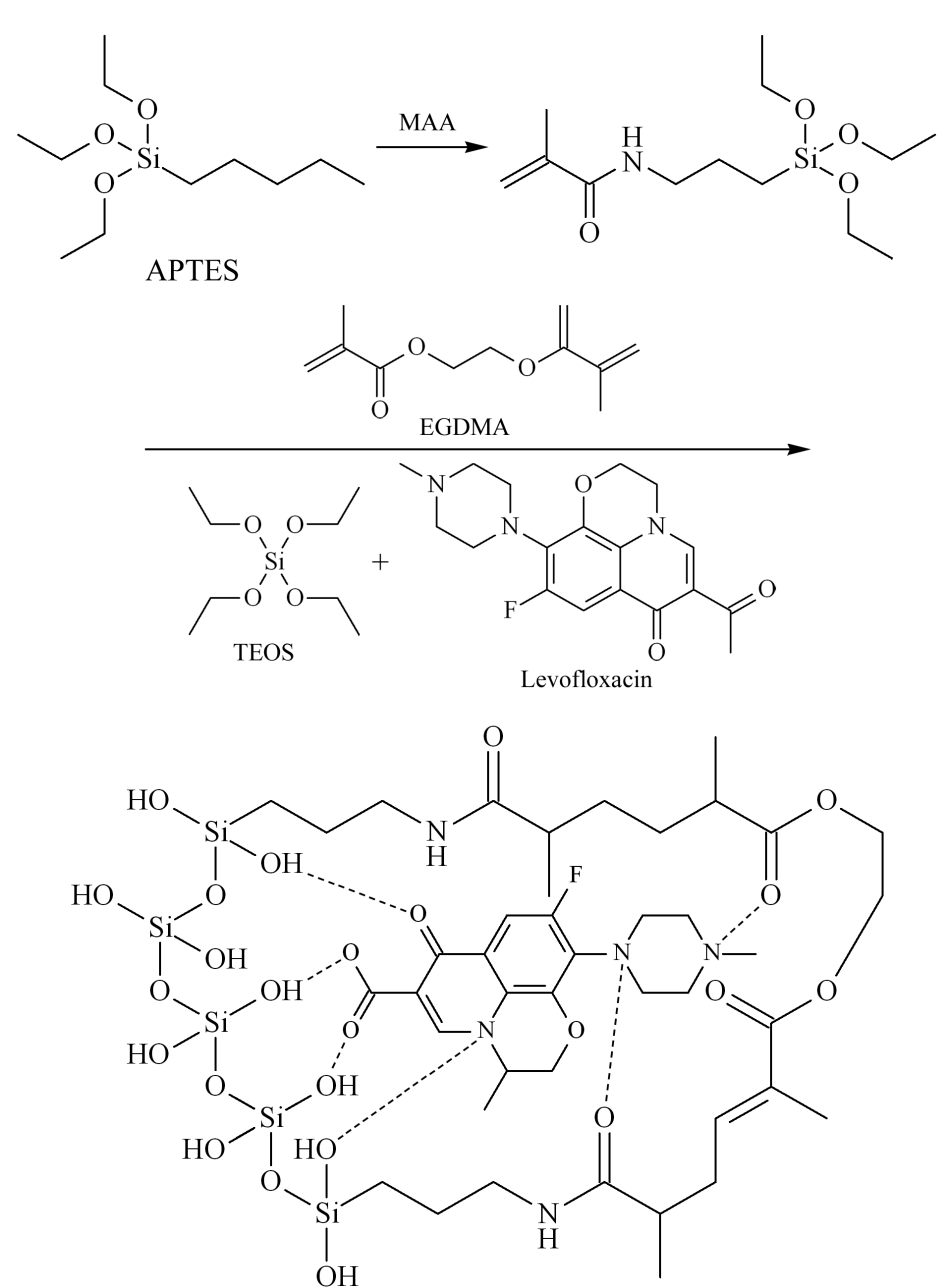
分子印迹材料合成过程示意图^[52]^

### 2.3 氧化石墨烯为载体

石墨烯是一种新型二维碳纳米平面结构,比表面积达2630 m^2^/g,具备理想吸附剂的特性,但存在团聚的趋势,有吸附机制单一等劣势,使用时多用含氧基团改性生成氧化石墨烯(graphene oxide, GO),从而提高石墨烯的性能,扩大其应用范围^[54,55]^。IL改性氧化石墨烯在固相萃取中的应用见表3。

**表3:** IL改性氧化石墨烯和磁性材料等在固相萃取中的应用

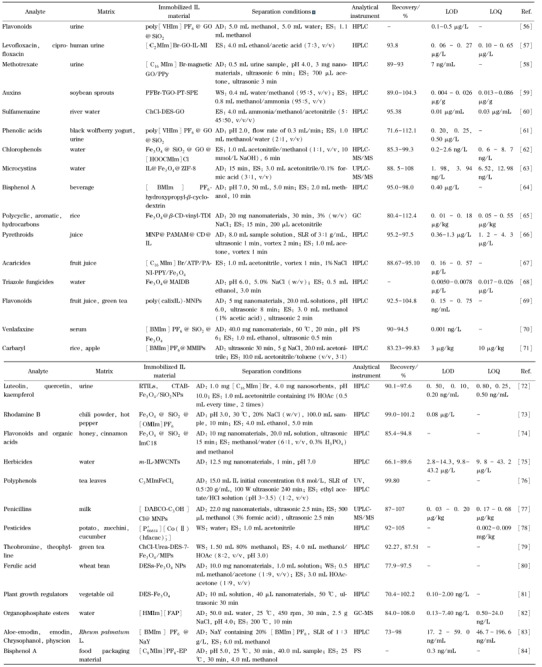

poly[VHIm ] PF_6_: poly( 1-vinyl-3-hexylimidazolium hexafluorophosphate); [C_2_MIm ] Br: 1-vinyl-3-ethylimidazolium bromide; [C_16_ MIm] Br: 1-hexadecyl-3-methylimidazolium bromide; PPy: polypyrrole; PFBr: 1-allyl-3-perfluorobenzyl-imidazolium bromide; TGO: thiol graphene oxide;[HOOCMIm] Cl: 1-carboxyl-3-methylimidazole chloride; MNP : magnetic nanoparti-cles; PAMAM: poly( amide amine ) dendrimers; ATP/PANI-PPY: attapulgite/ polyaniline-polypyrrole; MAIDB: 1-methyl-3-allylimidazolium bis( trifluoromethylsulfonyl ) imide copolymerize with divinylbenzene; MMIIPs: magnetic molecularly imprinted polymers; RTILs: l-hexadecyl-3-methylimidazolium bromide magnetic nanomaterials; CTAB: cetyltrimethylammonium brmide; [OMIm]PF_6_: 1-methyl-3-octylimidazolium hexafluorophosphate; Fe_3_O_4_ @SiO_2_ @ImC18: imidazole IL magnetic nanoparticles; MWCNTs: magnetic multi walled carbon nanotubes;C_3_MImFeCl_4_ : 1-propyl-3-methylimidazolium ferric chloride; [DABCO-C_3_OH]CI: N,N-dimethylaminoethanolamine; [$P^{+}_{66614}$][Co(Ⅱ) $(hfacac)^{-}_{3}$;] : quaternary phosphine hexafluoroacetyle-etone magnetic cobalt materials; [HMIm][FAP]: 1-hexyl-3-methylimidazolium trifluorophosphoric acid; NaY: Na-Y zeolite; [C_8_MIm ] PF_6_: 1-octyl-3-methylimidazolium tetrafluoroborate; EP: expanded perlite; FS: fluorescence spectroscopy; UV: ultraviole spectrophotometry.

Hou等^[56]^将GO包覆在SiO_2_上,通过原位阴离子交换反应制备聚IL-GO接枝SiO_2_材料聚1-乙烯基-3-己基咪唑六氟磷酸盐([VHIm]PF_6_@GO@SIL),固相萃取分离尿液中4种黄酮,即槲皮素、木犀草素、山柰酚和芹菜素,辅助高斯程序计算吸附剂和4个黄酮之间的相互作用能量;比较C18硅胶、聚[VHIm]PF_6_@Sil、GO、聚[VHIm]Br@GO@Sil和聚[VHIm]PF_6_@GO@Sil萃取4种黄酮的效果,发现虽然C18表面积达356.59 m^2^/g,明显大于改性后的硅胶材料,但聚[VHIm]PF_6_@GO@Sil依然具有更高的回收率和提取能力。Ma等^[57]^以环丙沙星和左氧氟沙星为模板剂,以1-乙烯基-3-乙基咪唑溴化铵([C_2_MIm]Br)为功能单体,以GO为核心材料,制备IL基双分子印迹聚合物涂层GO分子印迹整体柱,萃取分离尿液中环丙沙星和左氧氟沙星,并通过改变环丙沙星与左氧氟沙星含量比例、模板功能单体与交联剂比例,考察IL分子印迹整体柱的萃取效率和吸附容量。Hamidi等^[58]^通过Hummer’s方法合成GO,并制备了1-十六烷基-3-甲基咪唑溴盐([C_16_MIm]Br)功能化磁性氧化石墨烯/聚吡咯吸附剂IL磁性GO/PPy,用于固相分散微萃取尿液中的甲氨蝶呤;采用分子对接模拟计算技术,预测吸附剂与甲氨蝶呤之间的相互作用能量为8.35 kcal/mol,较大的结合能绝对值代表较大的吸附量,由此判断IL磁性GO/PPy对甲氨蝶呤具有更高的吸附强度。

管尖固相微萃取是一种小型化的样品预处理技术,以移液枪头为工具,两端放脱脂棉,其上端插入另外一个大号移液枪头,密封固定好,并用甲醇、水等溶剂活化装置,然后进行装样、淋洗、洗脱、收集淋洗液、检测等操作。Zhang等^[59]^采用硫醇-烯点击化学法,将1-烯丙基-3-全氟苄基-咪唑溴化铵(PFBr)接枝到TGO上,合成了一种新型IL硫醇-石墨烯氧化物(IL-TGO);比较了4种商用吸附剂HLB、C18、SCX和SiO_2_的吸附性能,用于吸附大豆豆芽中3种生长激素IAA、NAA和2,4-D,结果显示IL-TGO对3种生长素的回收率高于商业吸附剂,因IL-TGO可以通过氢键、静电作用、离子交换、*π-π*键相互作用吸附目标分子。刘玲玲等^[60]^制备出一种中性DES-GO材料,结合管尖固相微萃取技术,萃取河水中磺胺甲基嘧啶;通过考察淋洗剂和洗脱剂的种类、洗脱剂的体积等萃取因素,发现DES作为辅助性功能单体修饰后的GO比未修饰的GO具有更大的褶皱和比表面积,材料表面存在大量的亲水基团,能有效地改善吸附剂对磺胺甲基嘧啶的吸附能力。

Hou等^[61]^通过酰胺化反应、表面自由基链转移聚合和原位阴离子交换等方法,合成了一种环保型IL改性GO接枝SiO_2_新材料(PIL@GO@Sil),用于多种酚酸的固相萃取。与C18硅胶、GO@SIL、聚[VHIm]Br@SIL和聚[VHIm]PF_6_@SIL相比,用PIL改性的聚[VHIm]PF_6_@GO@SIL正电位更高,具有亲水性,对酸性化合物表现出更强的静电相互作用,对极性酚酸的回收率较高,因为GO的比表面积较大,能够增加IL的覆盖量和吸附位点,另外咪唑阳离子在GO表面上形成离域*π*电子体系,可通过*π-π*电荷作用与酚酸的芳香环相互作用,促使聚[VHIm]PF_6_@GO@SIL对极性化合物具有更好的萃取能力。Cai等^[62]^基于1-羧基-3-甲基咪唑氯盐([HOOCMIm]Cl),合成了一种平面GO磁性离子液体纳米材料(PGO-MILN),用于从环境水样中固相萃取2-氯酚、2,4-二氯苯酚、2,4,6-三氯酚、2,3,4,6-四氯苯酚和五氯苯酚;通过溶液pH、萃取时间、解吸因素等因素的考察,发现PGO-MILN对5种多氯苯酚具有良好的吸附性能,其吸附主要通过目标分子与纳米材料之间的空间效应、疏水作用和*π-π*键作用实现。

### 2.4 磁性材料为载体

磁性分散固相萃取以磁性或可磁化材料作为吸附剂,通过振荡、涡旋的方式使目标物与磁性吸附剂相结合,外加磁场实现目标物与基质磁性分离,最后用洗脱剂将目标物洗脱下来。该方法传质速率快,对大体积样品可快速吸附,能够解决传统吸附剂固液分离困难的问题,常见的磁性材料包括Fe、Fe_3_O_4_、MnFe_2_O_4_、MgFe_2_O_3_、磁性碳纳米管和磁性氧化石墨烯等,使用磁性材料时需要通过偶联剂、表面活性剂、高分子或天然生物大分子进行修饰改性,也可以通过SiO_2_和Ag进行碳包埋改性。

Fe_3_O_4_磁性粒子被研究最多,但其自身易被氧化,有强烈的聚集倾向,易发生沉淀,一般需经过表面包埋修饰后使用,例如用沸石材料包埋。吴怡秋等^[63]^通过多步合成法,先制备Fe_3_O_4_磁性粒子,再基于类沸石咪唑酯骨架材料制备Fe_3_O_4_@ZIF-8磁性微球,并基于1-丁基-3-甲基咪唑溴盐[BMIm]Br制备IL@Fe_3_O_4_@ZIF-8磁性复合纳米材料,结合UPLC-MS/MS测定水体中痕量微囊藻毒素;经扫描电镜和透射电镜分析,IL@Fe_3_O_4_@ZIF-8的核心为黑色的Fe_3_O_4_球体,外层包裹了IL和ZIF-8,材料表面有大量褶皱,从而增大了比表面积,提供了更多吸附位点;另外,磁性材料上咪唑基团同时存在于IL和ZIF-8材料上,有利于与微囊藻毒素的氨基和羧基通过静电吸引力相互结合,该方法的LOD值为0.004 μg/L,远低于《饮用水水质标准》规定的1 μg/L。

另外,可以由生物大分子*β*-CD进行接枝修饰Fe_3_O_4_磁性粒子。平文卉等^[64]^首先通过多步合成制成Fe_3_O_4_/羟丙基-*β*-CD聚合物纳米材料,然后采用浸渍法制备[BMIm]PF_6_负载环糊精磁性纳米材料,用于磁性固相萃取饮料中双酚A;研究了样品溶液pH、上样体积、吸附温度和时间等吸附条件对IL环糊精磁性材料吸附双酚A吸附率的影响。Boon等^[65]^研制了聚*β*-环糊精功能化IL固定化磁性纳米粒子(Fe_3_O_4_@*β*-CD-Vinyl-TDI),作为磁性吸附剂固相萃取大米中多环芳烃(PAHs);同等条件下,比较了Fe_3_O_4_纳米粒子、Fe_3_O_4_@*β*-CD-TDI、Fe_3_O_4_@*β*-CD-Vinyl-TDI、*β*-CD-Vinyl-TDI和TDI的提取能力,发现Fe_3_O_4_纳米粒子对PAHs的提取能力有限,Fe_3_O_4_@*β*-CD-Vinyl-TDI吸附剂的萃取效率明显优于Fe_3_O_4_@Vinyl-TDI,由于IL中含有-CH=CH_2_基和咪唑环,这些基团可以增强磁性材料与PAHs分析物的相互作用。Liu等^[66]^通过接枝法将聚酰胺-胺型树枝状高分子(PAMAM)接枝到磁性纳米粒子(MNPs)表面,合成磁芯树状大分子纳米粒子MNP@PAMAM,然后在超声作用下,将*β*-CD-O-Ts环糊精包埋于MNP@PAMAM表面形成MNP@PAMAM@CD,最后用浸渍法将纳米材料浸渍于1-辛基-3-甲基咪唑双(三氟甲基磺酰亚胺)([OMIm][Tf_2_N])中,制备出环糊精功能化磁性纳米材料(MNP@PAMAM@CD@IL),用于固相萃取果汁中拟除虫菊酯,研究发现MNP@PA-MAM@CD纳米材料具有八面体结构,表面平滑,被IL涂覆后,形状近似球形,表面粗糙;比较多种材料的萃取效率后发现,所制备的MNP@PAMAM@CD@IL对目标分子具有更高选择性,吸附容量更高。

凹凸棒石(ATP)是一种二维结晶水合硅酸镁非金属,具有共同的板条或纤维形态,含有四面体层。Yang等^[67]^基于ATP合成凹凸棒石/聚苯胺-聚吡咯/Fe_3_O_4_ (ATP/PANI-PPY/Fe_3_O_4_)纳米材料,并将[C_16_MIm]Br包覆于磁性纳米材料表面,建立磁性混合半胶束分散固相萃取方法,富集和分离果汁中3种杀螨剂;研究发现该IL纳米材料可以与目标分子之间形成强的相互作用,对杀螨剂具有较高的吸附效率。

Liu等^[68]^首先将1-甲基-3-烯丙基咪唑双(三氟甲基磺酰)亚胺与二乙烯基苯共聚,并采用自由基共聚法将其接枝于Fe_3_O_4_@SiO_2_@*γ*-MAPS表面,制成Fe_3_O_4_@MAIDB磁性材料(PFMA),用于固相萃取环境水中三唑类杀虫剂。参数优化后,PFMA磁性材料可以高效、快速地富集杀虫剂。Hu等^[69]^通过杯芳烃离子液体与硫醇改性的Fe_3_O_4_颗粒共聚,合成了一种新型磁性吸附剂聚杯芳烃IL-MNPs,用于分散固相萃取瓶装果汁和绿茶中痕量木犀草素、槲皮素、山柰酚、杨梅素和芹菜素;研究发现该吸附剂结构中的聚杯状空腔和刚性大环结构,能高效吸附目标黄酮,与其他方法相比,该方法具有较高的回收率和较低的检测限。

采用SiO_2_材料包埋修饰Fe_3_O_4_粒子的研究相对较多。Pirdadeh-Beiranvand等^[70]^采用IL [BMIm]PF_6_、双(三氟甲基磺酰亚胺盐)([BMPl]Tf_2_N)、1-乙基-3-甲基咪唑硫酸乙酯([EMIm][ETO]SO_3_)修饰Fe_3_O_4_纳米粒子,研制IL@SiO_2_@Fe_3_O_4_磁性吸附材料,用于固相萃取文拉法辛;扫描电镜图像显示,合成的Fe_3_O_4_纳米粒子具有近似球形的结构,尺寸在30~60 nm之间,但[EMIm][ETO]SO_3_不能包覆在Fe_3_O_4_纳米粒子表面,[BMPl]Tf_2_N@SiO_2_@Fe_3_O_4_改性吸附剂结构不稳定,解吸溶剂会导致IL从吸附剂表面被洗脱。Chen等^[71]^首先合成双键改性磁性纳米材料Fe_3_O_4_@SiO_2_,结合[BMIm]PF_6_和分子印迹技术,制备IL@MMIPs的磁性分子印迹聚合物,用于大米和苹果中氨基甲酸甲酯农药(CBR)的识别;实验表明,IL@MMIPs与磁性非印迹聚合物(IL@MNIPs)相比,具有良好的CBR选择性,更大的吸附容量和更短的平衡时间。He等^[72]^基于[C_16_MIm]Br和十六烷基三甲基溴化铵(CTAB),制备出2种Fe_3_O_4_/SiO_2_纳米复合材料,固相萃取尿液中微量黄酮,木犀草素、槲皮素和山柰酚;实验发现,[C_16_MIm]Br包埋的复合材料效果最好,回收率较高,LOD值较低,比已有方法更具优势。Chen等^[73]^选择3种疏水IL,即[BMIm]PF_6_、[HMIm]PF_6_和1-甲基-3-辛基咪唑六氟磷酸盐([OMIm]PF_6_),对Fe_3_O_4_@SiO_2_纳米粒子进行核壳结构包覆,制备Fe_3_O_4_@SiO_2_@IL材料,磁性固相萃取罗丹明B;同条件下对比实验,发现罗丹明B的吸附效率顺序为:Fe_3_O_4_<SiO_2_<Fe_3_O_4_@SiO_2_@[BMIm]PF_6_<Fe_3_O_4_@SiO_2_@[HMIm]PF_6_<Fe_3_O_4_@SiO_2_@[OMIm]PF_6_,所制备材料对罗丹明B的吸附效果主要取决于IL的疏水性,随着负载IL阳离子烷基链长度的增加,吸附率逐渐增加,Fe_3_O_4_@SiO_2_@[OMIm]PF_6_效率最高,目标物可以迅速吸附,用乙醇洗脱效果最好,其富集系数达25。Liu等^[74]^基于十八烷基咪唑离子液体,合成IL改性磁性纳米颗粒(Fe_3_O_4_@SiO_2_@ImC18),并选择12种化合物包括烷基苯、多环芳烃、黄酮和有机酸,用于评价了该新型吸附剂的MSPE性能。因所选的目标分子含有不同的活性基团,其吸附的初始浓度,以及洗脱液类型和体积各不相同。经过计算和拟合,所有吸附等温线均符合Langmuir模型,吸附过程中存在协同效应和竞争效应,多种相互作用共存,其作用差异顺序为:疏水相互作用>静电相互作用>*π-π*键和氢键相互作用;最终将该磁性材料用于实际样品蜂蜜中黄酮和肉桂中酚酸的磁性固相萃取,其中蜂蜜中杨梅素、槲皮素和木犀草素分别定量为0.17、0.50和0.12 μg/g,肉桂中咖啡酸和3,4-二甲氧基肉桂酸未检出,肉桂酸含量为25.1 μg/g。

Luo等^[75]^通过磁性纳米颗粒和咪唑IL修饰的碳纳米管自组装,制备出1-(3-氨丙基)咪唑IL改性的磁性多壁碳纳米管(m-IL-MWCNTs),固相萃取吸附环境水样中除草剂辛氧基苯氧基丙酸及其代谢产物;研究发现,该磁性材料主要基于*π-π*共轭及阴离子交换作用与除草剂发生作用,通过吸附剂的用量、样品溶液pH值、萃取时间和洗脱溶剂的优化,结合HPLC检测,发现该方法LOD和LOQ分别为2.8~14.3 μg/L和9.8~43.2 μg/L。Feng等^[76]^基于5种不同碳链长度的IL C_1~5_MImCl,合成5种黄褐色磁性咪唑IL材料(C*_n_*MIm FeCl_4_, *n*=1~5),用于磁选分离茶叶中茶多酚;结果发现,随着IL结构中烷基链长度的增加,磁性IL材料的极性降低,在水溶液中的溶解度和分散性能降低,同时阳离子的空间位阻增加,不利于磁性材料与目标分子之间发生相互作用;实验发现C_3_MImFeCl_4_具有最佳的萃取性能,以浓度为0.8 mol/L C_3_MImFeCl_4_磁性材料进行萃取,茶多酚的得率可达185.38 g/kg。Sahebi等^[77]^合成了*N*,*N*-二甲基乙醇胺(DABCO)基IL改性Fe_3_O_4_纳米粒子([DABCO-C_3_OH]Cl@MNPs),用于分散固相微萃取牛奶中5种青霉素,氨苄西林、苄青霉素、阿莫西林、苯唑西林和氯唑西林;SEM显微照片显示该材料粒径分布约为20 nm, TEM图像显示改性的IL包覆于Fe_3_O_4_纳米粒子的表面,能保护磁芯免受氧化和腐蚀,并易于与目标分析物产生较强亲和力。

Chatzimitakos等^[78]^基于季膦型IL([ $P_{66614}^{+}$ ])和六氟乙酰丙酮(hfacac)磁性金属盐,研制了5种IL磁性吸附材料:[ $P_{66614}^{+}$ ][Gd(Ⅲ)(hfacac ${)}_{4}^{-}$ ]、[ $P_{66614}^{+}$ ][Dy(Ⅲ)(hfacac ${)}_{4}^{-}$ ]、[ $P_{66614}^{+}$ ][Co(Ⅱ)(hfacac ${)}_{3}^{-}$ ]、[ $P_{66614}^{+}$ ][Mn(Ⅱ)(hfacac ${)}_{3}^{-}$ ]和[ $P_{66614}^{+}$ ][Ni(Ⅱ)(hfacac ${)}_{3}^{-}$ ],直接用于磁性固相萃取分离无预处理的高含水鲜蔬菜中存在的农药残留;研究发现磁性材料的黏度性质有助于与基体共混,其疏水性有利于农药的萃取。Co-和Mn-基材料,相比于Ni-基,用磁棒更容易回收,因Ni-具有较低的有效磁矩,另外,Co-材料为暗红色,Mn-材料为淡黄色,因前者萃取时更容易用眼睛识别,故选择Co-磁性材料进行后续研究。

DES与IL同样可以与目标分子生成稳定的氢键、离子电荷等相互作用力,因其制备简单,在分离中的应用较多。Li等^[79]^基于7种ChCl-尿素DESs和4种IL ([EMIm][Br]、[BMIm][Br]、[HMIm][Br]和[OMIm][Br]),结合分子印迹和磁选分离技术,制备IL磁性分子印迹聚合物材料,用于固相萃取绿茶中同分异构体可可碱和茶碱;对比DES基和IL基磁性材料,发现DES-Fe_3_O_4_/MIPs对2种异构体的回收率更高,其中DESs-7-Fe_3_O_4_/MIPs最高;与Fe_3_O_4_/MIPs相比,DESs-7-Fe_3_O_4_/MIPs的表面粗糙多孔、不规则,有团聚现象,DES-7的酰胺基嵌入在Fe_3_O_4_/MIPs纳米粒子中,静电和离子交换作用增强,ChCl和尿素的-NH包埋于纳米粒子表面,易于与可可碱和茶碱分子形成氢键,此外过量的DES-7被连接到材料的表面,作为辅助溶剂与目标物形成特定的结合位点,最终可可碱和茶碱的吸附量分别达4.87和5.07 mg/g。Qu等^[80]^通过化学共沉淀法制备Fe_3_O_4_纳米粒子(Fe_3_O_4_ NPs),并采用物质的量之比为1∶2的ChCl/乙醇DES对Fe_3_O_4_ NPs进行改性,用于从麦麸中磁性固相萃取阿魏酸,对比Fe_3_O_4_ NPs和DES-Fe_3_O_4_ NPs,发现后者对阿魏酸有更好的吸附效率,经过优化,阿魏酸的提取率达到了88.7%。Tan等^[81]^制备了DES-Fe_3_O_4_磁性复合材料,用于萃取食用植物油中的5种植物生长调节剂,吲哚-3-乙酸、脱落酸、噻唑仑、1-萘乙酸和氟氯芬隆;研究发现,萃取结束后溶液中Fe_3_O_4_能够被磁铁吸附,DES由于自身的重力沉降至试管底部,Fe_3_O_4_优先与DES结合在油基质中,促进了萃取后样品基质中DES的分离,实现了快速高效地从复杂的油基质中分离目标分子。

### 2.5 不锈钢丝、沸石及珍珠岩为载体

含FAP^—^阴离子的IL,疏水性和稳定性好。Shi等^[82]^通过直接浸涂,将1-己基-3-甲基咪唑三氟磷酸([HMIm][FAP])涂覆于不锈钢丝上,制成固相微萃取纤维,结合GC-MS用于分析环境水样中有机磷酸酯。经评价提取时间、提取温度、搅拌速度、离子强度、pH值和解析时间等因素后,发现该方法对水样中11种有机磷酸酯选择性和灵敏度与文献SPE-UPLC-MS/MS方法基本一致。

NaY沸石是一种由硅、铝、氧等组成的吸附剂,具有微孔结构均匀、热稳定性好、表面积大、吸附活性高等独特性能。Chen等^[83]^筛选了6种IL: [BMIm][PF_6_]、[C_6_MIm][PF_6_]、[C_8_MIm][PF_6_]、[BMIm][BF_4_]、[C_6_MIm][BF_4_]和[C_8_MIm][BF_4_],采用直接浸渍法制备IL-NaY吸附剂,用于吸附4种蒽醌;经参数优化,发现IL烷基链越长,与蒽醌作用越强,目标物洗脱越困难,6种材料对4种蒽醌的萃取率差别较大,对大黄素效果基本相当,其中[BMIm][PF_6_]和[C_6_MIm][PF_6_]吸附剂对大黄酚和大黄素-甲醚提取率更高,[BMIm][PF_6_]吸附剂对芦荟大黄素效果最好。

膨胀珍珠岩(EP)价格低廉且储量丰富,大多数珍珠岩含硅量大于70%,是一种密度约为32 g/L的超轻型材料,具有一定的吸附性能,同时可以浮在水面上,不需要离心就可以很方便地分离。Liu等^[84]^将[C_8_MIm][PF_6_]和EP在丙酮中涡旋混合2.5 h,制备了IL包覆的EP吸附剂,用于固相萃取食品包装材料中双酚A,结果表明双酚A可以快速吸附于IL-EP上,无需离心即可从水溶液中分离回收,同时以甲醇作为洗脱剂可显著增强双酚A的荧光。

## 3 结论与展望

IL含有羟基、氨基、羧基等活性基团,能够与有机分子生成稳定的*π-π*键、氢键、离子电荷等作用力,作用力越强,对目标分子识别选择性越好。为了更好地减少IL的流失,扩大其应用范围,学者进行了固定化研究,将IL固定到固体载体上制成复合材料,同时保留了IL和固体材料的特性,具有制备简单、富集效率高、吸附容量高、pH稳定性好、识别位点多、萃取选择性强、IL利用率高等优点,在固相萃取分离分析领域中的应用日益增多,已经应用于血样、尿样、水样、组织、中草药等复杂基质中生物大分子、金属离子、有机小分子等的萃取分离。现有的吸附剂多是基于咪唑IL设计,因为咪唑类结构稳定,易于发生作用,萃取过程中,或制成固相萃取柱使用,或作为分散剂使用,经一定条件吸附平衡后,再进行解析。同时,为了增强分散效果并减少分散剂的使用,需辅助一些辅助分散的手段,例如超声、搅拌、涡旋等。复合材料对于一些痕量有机污染物展现出良好的萃取效果,但对于基质比较复杂的样品如中草药活性成分,因干扰成分较多,其萃取效率和纯化程度并不理想。

关于IL固定化,有几个值得继续深入研究的方向。针对适合IL类型相对单一问题,需要更多的团队基于碳链长度、氨基酸等阴、阳离子结构类型,继续开发安全低毒、活性位点多的新型IL;针对基础理论研究相对薄弱的问题,需要更多学者对IL结构设计、固定化方法及应用领域继续进行深入的研究;针对生物样品分析的基质背景复杂的问题,需要将IL功能化,并与其他样品前处理技术相结合,构建采样、萃取、进样、分析一体化操作的在线分离分析模式,以提高分离的选择性和萃取效率。因此,根据IL的可设计性和载体的多样性,可以以功能化为导向,有针对性的设计出新型绿色的固定化离子液体材料,以展示其在各领域中更多的作用。
